# Lifetime exposure to smoking, epigenetic aging, and morbidity and mortality in older adults

**DOI:** 10.1186/s13148-022-01286-8

**Published:** 2022-05-28

**Authors:** Eric T. Klopack, Judith E. Carroll, Steve W. Cole, Teresa E. Seeman, Eileen M. Crimmins

**Affiliations:** 1 Leonard Davis School of Gerontology, University of Southern California, 3715 McClintock Ave., Los Angeles, CA 90089 USA; 2 Cousins Center for Psychoneuroimmunology, Department of Psychiatry & Biobehavioral Sciences, Jane & Terry Semel Institute for Neuroscience and Human Behavior, University of California, Los Angeles, Los Angeles, CA USA; 3 Cousins Center for Psychoneuroimmunology, Jane & Terry Semel Institute for Neuroscience and Human Behavior, University of California, Los Angeles, Los Angeles, CA 90095 USA; 4 Division of Geriatrics, David Geffen School of Medicine, University of California, Los Angeles, Los Angeles, CA 90095 USA

**Keywords:** Smoking, Epigenetic aging, Life course, Morbidity, Mortality

## Abstract

**Background:**

Cigarette smoke is a major public health concern. Epigenetic aging may be an important pathway by which exposure to cigarette smoke affects health. However, little is known about how exposure to smoke at different life stages affects epigenetic aging, especially in older adults. This study examines how three epigenetic aging measures (GrimAge, PhenoAge, and DunedinPoAm38) are associated with parental smoking, smoking in youth, and smoking in adulthood, and whether these epigenetic aging measures mediate the link between smoke exposure and morbidity and mortality. This study utilizes data from the Health and Retirement Study (HRS) Venous Blood Study (VBS), a nationally representative sample of US adults over 50 years old collected in 2016. 2978 participants with data on exposure to smoking, morbidity, and mortality were included.

**Results:**

GrimAge is significantly increased by having two smoking parents, smoking in youth, and cigarette pack years in adulthood. PhenoAge and DunedinPoAm38 are associated with pack years. All three mediate some of the effect of pack years on cancer, high blood pressure, heart disease, and mortality and GrimAge and DunedinPoAm38 mediate this association on lung disease.

**Conclusions:**

Results suggest epigenetic aging is one biological mechanism linking lifetime exposure to smoking with development of disease and earlier death in later life. Interventions aimed at reducing smoking in adulthood may be effective at weakening this association.

**Supplementary Information:**

The online version contains supplementary material available at 10.1186/s13148-022-01286-8.

## Background

Epigenetic clocks, or deoxyribonucleic acid methylation (DNAm) age, is a DNAm-based tool for assessing levels of DNAm related to aging health outcomes [1]. The first generation clocks (e.g., HorvathAge and HannumAge) are sets of DNAm sites that are highly correlated with chronological age [2, 3]. A second generation of clocks (e.g., GrimAge and PhenoAge) were trained instead on age-relevant biomarkers (e.g., serum creatinine, c-reactive protein), behaviors (e.g., smoking), and health outcomes, including mortality. Similarly, DunedinPoAm38 is a DNAm summary measure designed to capture the pace of aging. It was trained on change in biomarkers and health indicators over 12 years. These new generation DNAm aging measures have been shown to be strongly predictive of all-cause mortality, multiple morbidities, and frailty [4, 5]. There is also strong evidence they are sensitive to health behaviors like smoking [4, 6, 7].

### Exposure to smoke and epigenetic aging

Exposure to cigarette smoke increases risk for multiple morbidities—including cancer, cardiovascular disease, and lung disease—as well as mortality, and about one in five deaths in the US can be attributed to cigarette smoking [8]. One mechanism by which cigarette smoke exposure may affect health is through accelerated epigenetic aging—a DNAm-based measure of biological aging. Early life exposure to smoking, including prenatal maternal smoking, second-hand smoke exposure, and smoking in youth are associated with a range of important health outcomes, including lung function [9, 10], chronic obstructive pulmonary disease [11, 12], and cancer in adulthood [13–15]. Cigarette smoke causes damage to tissues. This damage accumulates, potentially driving accelerated epigenetic aging [6, 16–18]. Thus epigenetic aging may be an important pathway by which exposure to cigarette smoke affects health. However, little is known about how exposure to smoke at different life stages affects epigenetic aging, especially in older adults.

DNAm aging measures are objective indicators of accelerated aging that are relatively easy to collect and interpret and that have strong associations with social and behavioral predictors and health outcomes. However, there are a large number of unknowns that limit research utilizing these DNAm aging measures. These measures were all trained differently and have individual strengths and weaknesses. However, because studies typically utilize one DNAm aging measure at a time, it is unclear which measures are most useful as predictors of health outcomes and which are most affected by a given exposure. Because there has been relatively little life course research or research in older adults investigating the relationship between smoking and epigenetic age, it is unknown how early life and lifetime exposure to smoking and to second-hand smoke affect DNAm aging measures in older adults. To our knowledge, this is the first study investigating this association with second-generation DNAm aging measures in older adults.

### Current study

Given the potential health consequences of parental smoking, smoking in youth, and smoking in adulthood reviewed above, it is important to (1) explore how exposure to parental smoking affects smoking in youth and adulthood and (2) identify the physiological mechanisms by which smoke exposure across the life course affects chronic disease morbidity and mortality. Thus, this study builds on the large body of research described above to examine pathways by which parental smoking and smoking in youth and adulthood affect chronic illness morbidity and mortality. This study investigates how epigenetic aging measures as measures of biological aging mediate the associations between life course smoke exposure and chronic illness morbidity and mortality in a nationally representative sample of older adults (the 2016 Venous Blood Study (VBS) from the Health and Retirement Study (HRS)) [19, 20]. To our knowledge, this is the first study to do so. This research will help clarify when smoking cessation interventions may be most successful and will identify biological processes linking smoking to physical health.

## Results

### Descriptive statistics

Descriptive statistics are shown in Table 1. The weighted sample is 53% female and has a median age of 65 years. 73% of the sample is Non-Hispanic White, 12% is Non-Hispanic Black, 11% is Hispanic, and 4% is Non-Hispanic Other Race, 17% has less than 12 years of education, 31% has 12 years of education, 25% has 13–15 years of education, and 27% has 16 or more years of education. The sample had a median wealth of $157,000. More than a third of the sample is obese (37%), and 59% were non-drinkers.

**Table 1:** Descriptive statistics (N = 2978)

Variable	Mean/proportion	Standard Deviation	Range
Mortality	0.08		0 to 1
Cancer	0.18		0 to 1
High blood pressure	0.62		0 to 1
Lung disease	0.14		0 to 1
Heart disease	0.27		0 to 1
GrimAgeAdj	− 0.18	4.32	− 15.38 to 21.86
PhenoAgeAdj	0.08	6.76	− 22.56 to 41.44
DunedinPoAm38Adj	0.00	0.09	− 0.32 to 0.38
Pack years	11.64	18.80	0 to 138.94
Smoking in youth	0.20		0 to 1
Parent smoking			
Neither parent smoked	0.34		0 to 1
One parent smoked	0.42		0 to 1
Both parents smoked	0.24		0 to 1
Age	67.65	9.61	50 to 100
Race			
White, Not Hispanic	0.73		0 to 1
Black, Not Hispanic	0.12		0 to 1
Hispanic	0.11		0 to 1
Other, Not Hispanic	0.04		0 to 1
Gender			
Male	0.47		0 to 1
Female	0.53		0 to 1
Education			
0–11 years	0.17		0 to 1
12 years	0.31		0 to 1
13–15 years	0.25		0 to 1
16 + years	0.27		0 to 1
Log wealth	14.18	0.43	11.49 to 17.27
BMI			
Normal/underweight	0.26		0 to 1
Overweight	0.37		0 to 1
Obese 1	0.23		0 to 1
Obese 2	0.14		0 to 1
Alcohol use			
Non-drinker	0.59		0 to 1
1–4 drinks per time drinking	0.38		0 to 1
5 + drinks per time drinking	0.03		0 to 1

### Structural equation model results

**Fig. 1:**
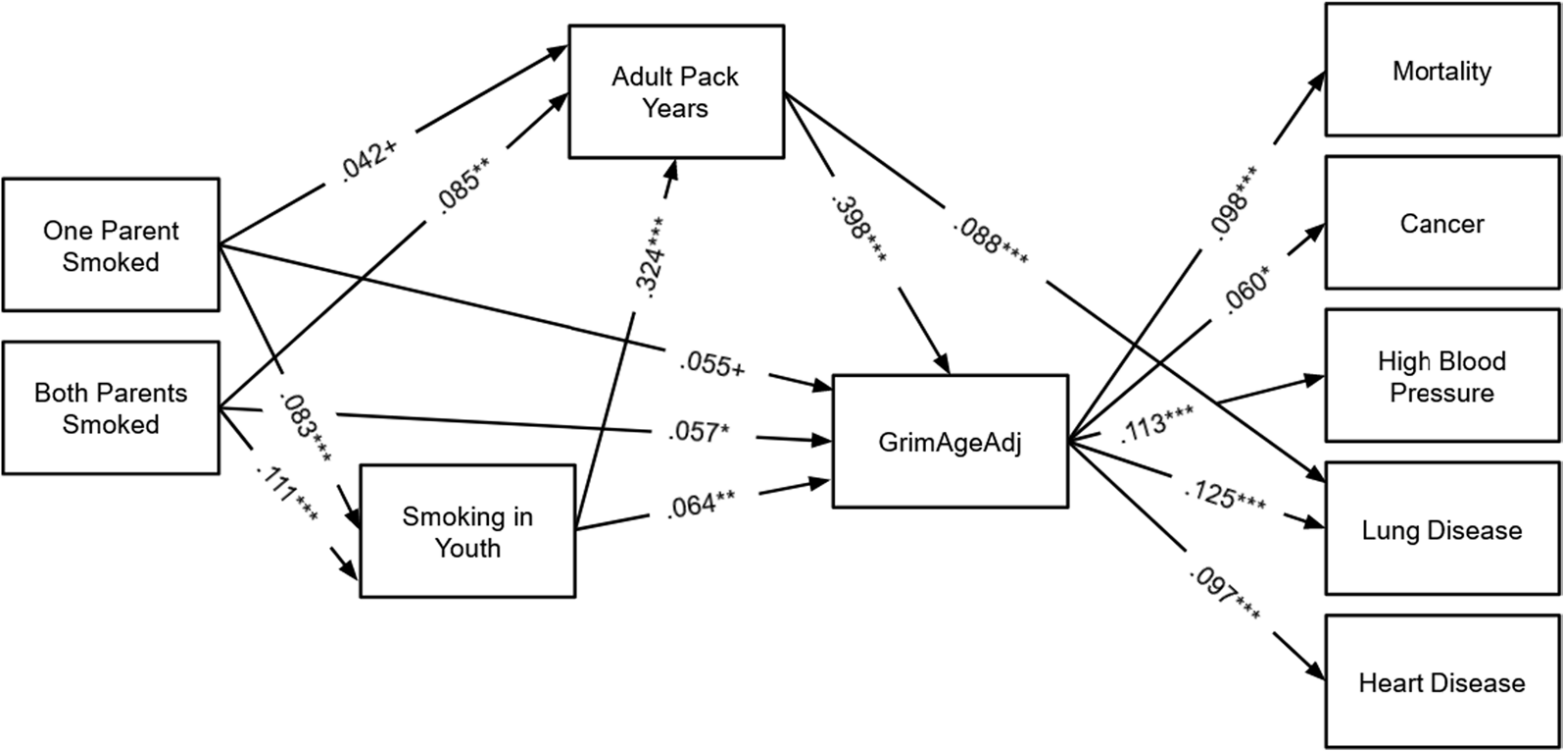
SEM Results for GrimAgeAdj. Note: *N* = 2978. Models are fully recursive; however, only significant theoretical pathways are shown. Total and indirect effects from this model are shown in Table 2, Panel B and Table 3, Panel B. ***p < .001, **p < .01, *p < .05

**Fig. 2:**
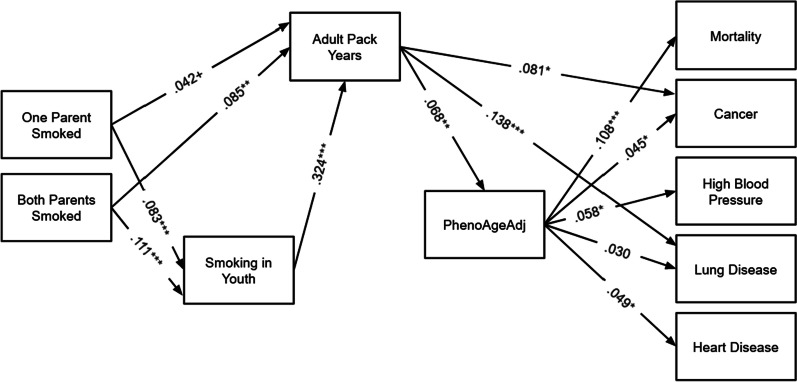
SEM Results for PhenoAgeAdj. Note: *N* = 2978. Models are fully recursive; however, only significant theoretical pathways are shown. Total and indirect effects from this model are shown in Table 2, Panel C and Table 3, Panel C. ***p < .001, **p < .01, *p < .05

**Fig. 3:**
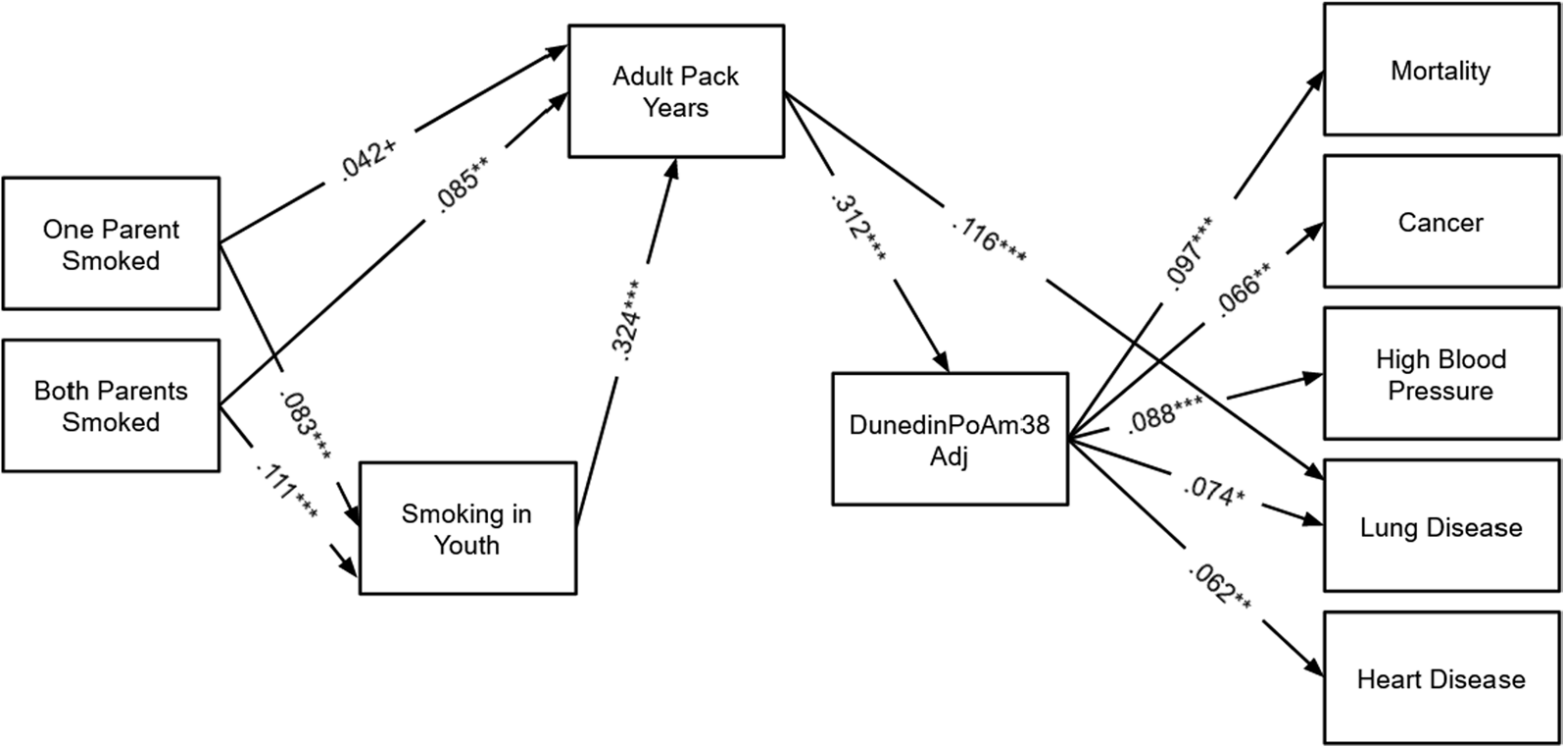
SEM Results for DunedinPoAm38Adj. Note: *N* = 2978. Models are fully recursive; however, only significant theoretical pathways are shown. Total and indirect effects from this model are shown in Table 2, Panel D and Table 3, Panel D. ***p < .001, **p < .01, *p < .05

Structural Equation Model (SEM) results are shown in Figs. 1, 2 and 3. These models are all fully recursive, but only significant and marginal hypothesized pathways are shown for ease of presentation.

Respondents who smoked in youth and who had two parents who smoked tended to have more adult pack years. That is, respondents who smoked in youth were predicted to smoke about 15 more pack years of cigarettes in adulthood. Respondents were predicted to smoke about 4 more pack years of cigarettes in adulthood if both of their parents smoked. Parent smoking was associated with significantly higher probability of smoking in youth.

Turning now to results for GrimAgeAdj (Fig. 1), adult pack years, smoking in youth, and having two parents who smoked were significantly associated with greater GrimAgeAdj. For each additional pack year, participants were expected to have a GrimAgeAdj about 0.09 years greater than similar peers. Participants who smoked in youth were expected to have a GrimAgeAdj about 0.7 years older than similar peers who did not. If both of a participant’s parents smoked, they were expected to have a grim age 0.6 years greater than similar peers for whom neither parent smoked. GrimAgeAdj significantly predicted mortality, and having cancer, high blood pressure, lung disease, and heart disease. A one year higher GrimAgeAdj was associated with an increased risk of each of these outcomes.

PhenoAgeAdj (Fig. 2), alternatively, was only significantly predicted by adult pack years, such that each additional pack year was associated with a 0.03 year greater PhenoAgeAdj. PhenoAgeAdj significantly predicted all health outcomes in the model except lung disease. A one year higher PhenoAgeAdj was associated with an increased risk of mortality, cancer, high blood pressure, and heart disease.

Finally, DunedinPoAm38Adj (Fig. 3) was only significantly associated with adult pack years. Each additional pack year was associated with a pace of biological aging of 0.001 years per chronological year faster than similar peers with 1 fewer pack year. DunedinPoAm38 was significantly associated with all five health outcomes. A DunedinPoAm38 pace of aging increase of 1 year per chronological year was associated with an increased risk of mortality, cancer, high blood pressure, lung disease, and heart disease.

### Mediation results

Turning to mediation results (Tables 2, 3), the total effects of adult pack years on cancer and lung disease were significant and the total effect of smoking in youth on lung disease was significant. No other total effects were significant. Because none of the total effects of parental smoking on health outcomes were significant, we do not focus on those indirect effects here.

**Table 2:** Total and indirect effects of adult pack years on health outcomes (N = 2978)

Outcome	Total effect	95% confidence interval	
*Panel A. Total effects of adult pack years on health outcomes*			
Mortality	0.0006	0.0000	0.0012
Cancer	0.0019	0.0003	0.0036
High blood pressure	0.0009	− 0.0001	0.0018
Lung disease	0.0029	0.0020	0.0038
Heart disease	0.0013	− 0.0001	0.0026

Outcome	Mediator(s)	Indirect effect	95% confidence interval	
*Panel B. Indirect effects of adult pack years on health outcomes—GrimAgeAdj*				
Mortality	GrimAgeAdj	0.0005	0.0003	0.0008
Cancer	GrimAgeAdj	0.0006	0.0001	0.0010
High blood pressure	GrimAgeAdj	0.0012	0.0007	0.0017
Lung disease	GrimAgeAdj	0.0011	0.0006	0.0015
Heart disease	GrimAgeAdj	0.0010	0.0005	0.0015
*Panel C. Indirect effects of adult pack years on health outcomes—PhenoAgeAdj*				
Mortality	PhenoAgeAdj	0.0001	0.0000	0.0002
Cancer	PhenoAgeAdj	0.0001	0.0000	0.0002
High blood pressure	PhenoAgeAdj	0.0001	0.0000	0.0003
Lung disease	PhenoAgeAdj	0.0000	0.0000	0.0001
Heart disease	PhenoAgeAdj	0.0001	0.0000	0.0002
*Panel D. Indirect effects of adult pack years on health outcomes—DunedinPoAm38Adj*				
Mortality	DunedinPoAm38Adj	0.0004	0.0002	0.0006
Cancer	DunedinPoAm38Adj	0.0005	0.0001	0.0008
High blood pressure	DunedinPoAm38Adj	0.0007	0.0003	0.0012
Lung disease	DunedinPoAm38Adj	0.0005	0.0001	0.0009
Heart disease	DunedinPoAm38Adj	0.0005	0.0001	0.0009

**Table 3:** Total and indirect effects of smoking in childhood on health outcomes (N = 2978)

Outcome	Total effect	95% confidence interval	
*Panel A. Total effects of smoking in youth on health outcomes*			
Mortality	0.0012	− 0.0265	0.0284
Cancer	0.0323	− 0.0193	0.0848
High blood pressure	0.0071	− 0.0452	0.0596
Lung disease	0.0749	0.0282	0.1213
Heart disease	0.0249	− 0.0292	0.0788

Outcome	Mediator(s)	Indirect effect	95% confidence interval	
*Panel B. Indirect effects of smoking in youth on health outcomes—GrimAgeAdj*				
Mortality	GrimAgeAdj	0.0042	0.0011	0.0076
Cancer	GrimAgeAdj	0.0042	0.0003	0.0101
High blood pressure	GrimAgeAdj	0.0092	0.0021	0.0184
Lung disease	GrimAgeAdj	0.0080	0.0018	0.0160
Heart disease	GrimAgeAdj	0.0075	0.0017	0.0151
Mortality	Adult Pack Years	0.0008	− 0.0093	0.0110
Cancer	Adult Pack Years	0.0207	− 0.0084	0.0527
High blood pressure	Adult Pack Years	− 0.0054	− 0.0214	0.0112
Lung disease	Adult Pack Years	0.0284	0.0136	0.0436
Heart disease	Adult Pack Years	0.0041	− 0.0149	0.0229
Mortality	Adult Pack Years—> GrimAgeAdj	0.0084	0.0042	0.0128
Cancer	Adult Pack Years—> GrimAgeAdj	0.0084	0.0013	0.0155
High blood pressure	Adult Pack Years—> GrimAgeAdj	0.0184	0.0110	0.0264
Lung disease	Adult Pack Years—> GrimAgeAdj	0.0161	0.0091	0.0235
Heart disease	Adult Pack Years—> GrimAgeAdj	0.0150	0.0082	0.0222
*Panel C. Indirect effects of smoking in youth on health outcomes—PhenoAgeAdj*				
Mortality	PhenoAgeAdj	− 0.0007	− 0.0044	0.0024
Cancer	PhenoAgeAdj	− 0.0005	− 0.0035	0.0016
High blood pressure	PhenoAgeAdj	− 0.0007	− 0.0052	0.0026
Lung disease	PhenoAgeAdj	− 0.0003	− 0.0027	0.0011
Heart disease	PhenoAgeAdj	− 0.0006	− 0.0040	0.0022
Mortality	Adult Pack Years	0.0080	− 0.0012	0.0172
Cancer	Adult Pack Years	0.0285	0.0031	0.0565
High blood pressure	Adult Pack Years	0.0122	− 0.0025	0.0281
Lung disease	Adult Pack Years	0.0445	0.0299	0.0601
Heart disease	Adult Pack Years	0.0185	− 0.0022	0.0393
Mortality	Adult Pack Years—> PhenoAgeAdj	0.0016	0.0003	0.0034
Cancer	Adult Pack Years—> PhenoAgeAdj	0.0011	0.0001	0.0028
High blood pressure	Adult Pack Years—> PhenoAgeAdj	0.0016	0.0001	0.0040
Lung disease	Adult Pack Years—> PhenoAgeAdj	0.0007	− 0.0002	0.0021
Heart disease	Adult Pack Years—> PhenoAgeAdj	0.0013	0.0001	0.0032
*Panel D. Indirect effects of smoking in youth on health outcomes—DunedinPoAm38*				
Mortality	DunedinPoAm38Adj	0.0008	− 0.0032	0.0037
Cancer	DunedinPoAm38Adj	0.0008	− 0.0031	0.0054
High blood pressure	DunedinPoAm38Adj	0.0013	− 0.0047	0.0079
Lung disease	DunedinPoAm38Adj	0.0009	− 0.0032	0.0058
Heart disease	DunedinPoAm38Adj	0.0009	− 0.0036	0.0050
Mortality	Adult Pack Years	0.0028	− 0.0063	0.0120
Cancer	Adult Pack Years	0.0220	− 0.0035	0.0495
High blood pressure	Adult Pack Years	0.0022	− 0.0128	0.0180
Lung disease	Adult Pack Years	0.0374	0.0235	0.0521
Heart disease	Adult Pack Years	0.0121	− 0.0086	0.0325
Mortality	Adult Pack Years—> DunedinPoAm38Adj	0.0065	0.0035	0.0098
Cancer	Adult Pack Years—> DunedinPoAm38Adj	0.0072	0.0020	0.0128
High blood pressure	Adult Pack Years—> DunedinPoAm38Adj	0.0112	0.0050	0.0182
Lung disease	Adult Pack Years—> DunedinPoAm38Adj	0.0075	0.0018	0.0135
Heart disease	Adult Pack Years—> DunedinPoAm38Adj	0.0074	0.0022	0.0132

In the GrimAgeAdj model (Table 2, Panel B and Table 3, Panel B; shown in Fig. 1), all of the indirect paths from adult pack years to health outcomes were significant. That is, although the total effects of adult pack years were not all significant, each additional adult pack year was associated with an increased probability of mortality, cancer, high blood pressure, lung disease, and heart disease mediated by GrimAgeAdj. GrimAgeAdj mediated 32% of the total significant effect of adult pack years on cancer and 38% of the total significant effect of adult pack years on lung disease. Additionally, GrimAgeAdj mediated 11% of the total effect of smoking in youth on lung disease, and the path smoking in youth—> adult pack years—> GrimAgeAdj—> lung disease mediated 21% of the total effect of smoking in youth on lung disease. That is, smoking in youth affected probability of lung disease in later life partly because smoking in youth was directly associated with accelerated GrimAgeAdj aging and partly because smoking in youth was tied to smoking in adulthood, which was associated with accelerated GrimAgeAdj.

Results for PhenoAgeAdj (Table 2, Panel C and Table 3, Panel C; as shown in Fig. 2) are less supportive. None of the indirect effects from adult pack years to health outcomes mediated by PhenoAgeAdj were significant, and none of the indirect effects from smoking in youth to lung disease mediated by PhenoAgeAdj were significant. Thus, PhenoAgeAdj does not appear to be a meaningful mediator of the association between smoke exposure and health outcomes investigated here.

Results from the model for DunedinPoAm38Adj (Table 2, Panel D and Table 3, Panel D; shown in Fig. 3) were similar to those for GrimAgeAdj. That is, all of the indirect paths from adult pack years to health outcomes were significant. Again, although the total effects of adult pack years were not all significant, each additional adult pack year was associated with an increased probability of mortality, cancer, high blood pressure, lung disease, and heart disease mediated by DunedinPoAm38Adj. DunedinPoAm38Adj mediated 26% of the total significant effect of adult pack years on cancer and 17% of the total significant effect of adult pack years on lung disease. DunedinPoAm38Adj did not significantly directly mediate total effect of smoking in youth on lung disease, but the path smoking in youth—> adult pack years—> DunedinPoAm38Adj—> lung disease mediated 10% of the total effect of smoking in youth on lung disease. That is, smoking in youth was associated with probability of lung disease in later life partly because smoking in youth was tied to smoking in adulthood, which was associated with accelerated DunedinPoAm38Adj.

### Additional analyses

We also estimated the same model with the first generation clocks HorvathAgeAdj and HannumAgeAdj (not shown). HorvathAgeAdj was not significantly associated with any of the health outcomes investigated here and was only significantly associated with having two parents who smoked, though in the wrong direction (*b* = − 1.131, *p* = 0.003). HannumAgeAdj was significantly associated with cancer risk (*b* = 0.007, *p* = 0.001) and high blood pressure (*b* = 0.005, *p* = 0.016), but was not significantly associated with any of the life course smoking variables. Thus, these first generation clocks do not appear to be plausible mediators of the association between life course smoking exposure and the health outcomes examined here.

It is also possible to assess each of the components of GrimAge separately. We estimated the same model as above with each of the components (Additional file 1: Figs. S1–S8). Components were on very large scales, so each was divided by 1000 to reduce variance and ease SEM estimation. Because each component was on a different scale, we present standardized coefficients to make estimates comparable. Only DNAm estimated pack years was significantly associated with having one parent who smoked, two parents who smoked, or smoking in youth. DNAm estimated adrenomedullin, cystatin-C, growth/differentiation factor-15, pack years, plasminogen activator inhibitor type 1, and tissue inhibitor of metalloproteinase 1 (TIMP-1) were all significantly associated with adult pack years. The indirect effects of smoking in youth and adult pack years on lung disease appear to be mostly driven by DNAm surrogate pack years which explained 20.52% and 34.96% of those total effects, respectively. DNAm surrogate TIMP-1 is the only component that significantly mediates the association between adult pack years and cancer risk, explaining about 5% of this total association.

## Discussion

Past research suggests DNAm aging measures represent a plausible biological pathway by which exposure to cigarette smoke affects mortality and multiple chronic morbidities, including cancer, high blood pressure, lung disease, and heart disease. However, little is known about how exposure to smoking across the life course affects DNAm aging measures and these health outcomes. This study builds on this past research by utilizing a nationally representative sample of US adults over age 50 to test whether smoking exposure by three potentially critical vectors (parental smoking, smoking in youth, and adult pack years) affects four chronic disease morbidities and mortality and whether these effects are mediated by three second-generation DNAm aging measures. Results showed all three DNAm aging measures were significantly affected by adult pack years and all three significantly predicted the health outcomes examined here (except PhenoAgeAdj was not associated with lung disease). Additionally, GrimAgeAdj was significantly associated with smoking in youth and having two parents who smoked. GrimAgeAdj and DunedinPoAm38Adj were both important mediators of the total effects of adult smoking on cancer and lung disease, and GrimAgeAdj played a role in the link between smoking in youth and lung disease in later life.

The epigenome in general [21–24] and these aging measures in particular [6, 16–18] are highly affected by smoking exposure. By establishing when in the life course smoking exposure affects these measures and to what degree, the current work helps to clarify how DNAm aging measure are associated with life course smoking exposure. Results suggest GrimAge is most sensitive to life course smoking exposure, as it is independently affected by adult pack years, smoking in youth, and parental smoking. GrimAge and DunedinPoAm38 both appear to be plausible pathways connecting adult smoking to cancer and lung disease. Additionally, GrimAge significantly mediated the association between smoking in youth and lung disease. These findings are consistent with past research showing GrimAge and DunedinPoAm38 are affected by smoking more strongly than PhenoAge [6, 25]. The DNAm surrogate pack years component appeared to be the main reason GrimAgeAdj mediated the associations between smoking in youth and adult pack years and lung disease, suggesting epigenetic aging measures that directly incorporate smoking (e.g., GrimAge) may more fully capture life course smoke exposure. The DNAm surrogate TIMP-1 component appeared to be the main reason GrimAgeAdj mediated the association between adult pack years and cancer, consistent with past research suggesting TIMP-1 plays an important role in cell proliferation in cancerous tissue [26].

These results also suggest damage caused by smoking early in life increases risk of lung disease via several pathways. First, people who smoke in youth are also likely to smoke in adulthood and are thus continuously exposed to smoking-related risk. Second, smoking in youth appears to directly affect adult GrimAge, independent of later smoking behavior. Thus, GrimAge may capture permanent damage caused by smoking early in life. Further longitudinal research is needed investigating how smoking during developmental critical periods differentially affects later life biological aging. Additional research is needed investigating the effect of smoking cessation on epigenetic aging. Early investigations suggest cessation can substantially reduce the epigenetic aging and telomere attrition associated with smoking [27, 28].

The current study has some key limitations. Smoking in youth and pack years for former smokers were assessed using a retrospective self-report and may be biased by recall and social desirability. Our measures of chronic disease morbidity were self-reported. Future work should validate our results in a well characterized clinical population.

Despite these limitations, these results provide evidence that accelerated DNAm aging may be a plausible biological mechanism linking smoking in youth to lung disease later in life and smoking in adulthood to cancer and lung disease. These results suggest a substantial portion of the lung disease risk of smoking in youth is associated with accelerated DNAm aging; however, a larger portion was explained by pathways involving adult smoking. Though early life smoking may cause some permanent damage, cessation in adulthood reduces a large proportion of the risk. Thus, interventions focused on prevention of early life smoking are essential, and efforts focused on cessation in adulthood are also important.

## Methods

### Sample

We utilize data from the DNA methylation subsample from the Health and Retirement Study 2016 Venous Blood Study (*N* = 4018) [29]. A certified phlebotomist collected 50.5 mL of blood from consenting participants within 4 weeks of the HRS core interview (if possible) in the participants’ homes. DNA for DNAm analysis was extracted from an ethylenediamine tetraacetic acid (EDTA) whole blood tube. DNAm analysis was conducted by the Clinical Laboratory Improvement Amendments of 1988 (CLIA) certified Advanced Research and Diagnostic Laboratory at the University of Minnesota. Detailed methods for this sample are published elsewhere [19, 20]. This sample was developed to be representative of the U.S. population over age 50 when weighted.

2978 participants were included in the current study due to missingness in independent variables. 23 participants were missing on the pack years measurement, 43 were missing on the smoking in childhood measure, 941 were missing on the parental smoking measure, 17 were missing on at least one health outcomes, and 76 were missing on at least one control variable.

### Measures

#### Chronic conditions and mortality

We examine morbidity in four chronic conditions associated with smoking [30] (viz., cancer, high blood pressure, lung disease, and heart disease) and mortality. Participants were asked whether or not a doctor had told them they had cancer or a malignant tumor of any kind except skin cancer, high blood pressure or hypertension, chronic lung disease except asthma such as chronic bronchitis or emphysema, or heart attack, coronary heart disease, angina, congestive heart failure, or other heart problems at the time of the interview. If responses were missing from the 2018 interview, 2016 responses were used. Mortality was assessed by identifying participants who were known to be deceased as of 2020.

#### DNAm aging measures

We utilize three DNAm aging measures that have been widely applied in past research (viz., GrimAge, PhenoAge, and DuneinPoAm38). GrimAge was trained on 7 DNAm surrogates of plasma proteins associated with mortality and pack years [31]. PhenoAge was trained on 9 blood-based markers of immune and tissue function [32]. DunedinPoAm38 was trained on changes in biomarkers and health indicators related to healthy aging [33]. GrimAge and PhenoAge are both scaled in years and are meant to capture epigenetic age at the time of measurement. DunedinPoAm38 is meant to capture pace of aging and is scaled in years of epigenetic aging per chronological year. Because it focuses on current pace of aging, DunedinPoAm38 may be more sensitive to recent smoking behaviors; whereas, the second generation clocks were designed to capture current DNAm age and may be more sensitive to early life and other life course exposures. These three measures were trained in separate samples with different ages groups represented. GrimAge was first trained in the Framingham Heart Study offspring cohort (ages 53–73), PhenoAge in the InCHIANTI cohort (ages 21–100), and DunedinPoAm38 in the Dunedin Study cohort (a birth cohort with clocks estimated at age 38). It is possible that measures trained in older samples may be better able to capture epigenetic aging in the older American HRS sample.

#### Smoking and smoke exposure

Life course smoke exposure as assessed using self-reports of parental smoking, smoking in youth, and smoking pack years in adulthood. More detailed information about these measures is available in the Additional file 1.

#### Controls

In all models in current study, we control for age, race, and gender. We also control for education coded as 0–11 years, 12 years, 13–15, or 16+ years (reference group), wealth (log transformed), BMI categorized as 25–29.99, 30–34.99, 35+, or < 25 (reference group), and alcohol use categorized as 1–4 drinks per day drinking, 5+ drinks per day drinking, or non-drinker (reference group).

### Plan of analysis

Three structural equation models (SEMs) were estimated regressing chronic disease morbidities and mortality on each of the DNAm aging measures, adult pack years, smoking in youth and parent smoking, adult pack years regressed on smoking in youth and parent smoking, and smoking in youth regressed on parent smoking. Because the DNAm aging measures covary highly with the control variables, we regressed each DNAm aging measure on the controls and computed the residuals. These residualized DNAm aging measures are used in the analyses. We are thus able to adjust for these potential confounders and avoid issues associated with multicollinearity. In tables, figures, and the text, all DNAm aging measures have the suffix “Adj” appended to their name to indicate they are adjusted for age, race, gender, education, wealth, BMI, and alcohol use. To facilitate comparisons among effects, standardized coefficients are shown in the figures. For main effects, both standardized and unstandardized coefficients are noted in the text. Survey weights and strata were applied from the HRS tracker file. Participants missing Venous Blood Study-specific weights were assigned their 2016 core weight. Having a large number of parameters relative to the number of clusters can cause estimation problems in SEM. We therefore estimated models with all variables residualized using the same process as described for DNAm aging measures above. These models produced highly similar results with a nearly identical pattern of significance.

Because indirect effects may not be normally distributed, common significance tests that assume a normal distribution can be biased. Therefore, indirect effects were assessed using the Monte Carlo method. In this method, effects are estimated for the sample, a sampling distribution of the product of the independent and mediating variable are generated based on 1000 random samples with population values equal to the sample values, and lower and upper confidence intervals are generated based on this distribution [34]. This method has been shown to be comparable to other asymmetric methods and is appropriate for complex survey data [35]. Thus, indirect effects were calculated based on the SEM models. All analyses were conducted in R 4.1.1 [36] using the survey [37], lavaan [38], lavaan.survey [39], and semTools [40] packages. R code to reproduce the figures is available at https://github.com/etklopack/lifetime_smoking_epigenetic_aging.

## Supplementary Information

**Additional file 1:** Supplementary Information.

## Data Availability

All data used in the current study are publicly available at https://hrs.isr.umich.edu/data-products.
