# Supplementary material for: Lifetime exposure to smoking, epigenetic aging, and morbidity and mortality in older adults
Source: Clin Epigenetics. 2022 May 28;14:72. doi: 10.1186/s13148-022-01286-8 (PMC9148451; doi:10.1186/s13148-022-01286-8)

## **Additional file 1**

## **Lifetime Exposure to Smoke, Morbidity, and Mortality**

### **Parental Smoking**

There is evidence from past research that parental smoking is associated with adult morality, cardiovascular disease, cancer[^1^](https://www.zotero.org/google-docs/?G7DY65), and lung disease[^2^](https://www.zotero.org/google-docs/?kQYTFN). Parents who smoke may expose their child to second-hand smoke, which has been linked to all the health issues listed above[^3^](https://www.zotero.org/google-docs/?XN7m1W). Past evidence suggests that environmental smoke exposure in childhood affects lung development, leading to respiratory problems throughout life[^4^](https://www.zotero.org/google-docs/?GuIBuR). Additionally, evidence suggests that children model their parents’ health behaviors. That is, children are more likely to smoke if their parents smoke[^5^](https://www.zotero.org/google-docs/?11SPjC). Parents who smoke may increase their child’s risk of smoking and thus exposure to carcinogens and other toxins.

### **Smoking in Youth and Adulthood**

Smoking during youth may affect lung growth and functioning, leading to long-lasting damage that can affect lung health in adulthood[^2^](https://www.zotero.org/google-docs/?drHOQ1). Smoking in youth causes cellular and molecular damage that amplify the health risks of smoking in adulthood[^6^](https://www.zotero.org/google-docs/?SQRDZW). Additionally smoking in youth is strongly predictive of smoking in adulthood[^5,7^](https://www.zotero.org/google-docs/?SpIJgD). Smoking in youth may set individuals on a trajectory of increased tobacco use throughout life, greatly increasing their exposure to carcinogens and other toxins and increasing their risk of morbidity and mortality. Additionally, there is extensive evidence linking smoking in adulthood to cardiovascular disease, lung disease, cancer, and mortality[^7^](https://www.zotero.org/google-docs/?JNJoGQ).

**Additional Information about Smoking Variables**

**Parent smoking.** Participants were asked if their parents or guardians smoked during their childhood as part of the 2015 and 2017 life history mail survey. Responses are categorized both parents smoked, one parent smoked, or neither parent smoked. If respondents only reported on one parent, the other parent’s status was assumed to be non-smoking.

**Smoking in youth.** As part of the core survey from 2008-2016, participants were asked if they regularly smoked cigarettes in grade school or high school, with regularly defined as: ”at least one cigarette a day for most days of the week, for six months or more”. If responses were missing for 2008, values from 2010 were used and so forth through 2016.

**Adult pack years.** Respondents reported detailed information about when they began and quit smoking cigarettes, as well as how much they smoked while they were smoking. A respondent's earliest age reported for starting and latest age for cessation were used to calculate number of years of smoking if the respondent started after age 18. If a respondent began before age 18, age 18 and the latest age reported for cessation were used to calculate number of years of smoking. Thus, this measure only captures smoking after age 17. Packs of cigarettes smoked per day was calculated from both the average of the reported number of cigarettes per day at each wave for each individual, and the maximum number of cigarettes smoked during the time in which the individual reported smoking the most.

Around 10,123 ever smokers (44%) had at least one missing value for calculating pack-years. These individuals included ever smokers with no reported age of start, former smokers with unknown age of cessation, and ever smokers who did not report the number of cigarettes per day. Since this was a large portion of the population, missing values were imputed using the average age of starting, age of cessation, and number of cigarettes per day calculated from the 12,585 ever smokers who had complete data. For more information about this measure, see Haghani (2020)[^8^](https://www.zotero.org/google-docs/?JfXy6c).

[1. Clarke MA, Joshu CE. Early Life Exposures and Adult Cancer Risk. *Epidemiol Rev*. 2017;39(1):11-27. doi:10.1093/epirev/mxx004](https://www.zotero.org/google-docs/?0AOe1V)

[2. Grant T, Brigham EP, McCormack MC. Childhood Origins of Adult Lung Disease as Opportunities for Prevention. *J Allergy Clin Immunol-Pract*. 2020;8(3):849-858. doi:10.1016/j.jaip.2020.01.015](https://www.zotero.org/google-docs/?0AOe1V)

[3. U.S. Department of Health and Human Services, Office of the Surgeon General. *The Health Consequences of Involuntary Exposure to Tobacco Smoke: A Report of the Surgeon General*.; 2006. https://www-ncbi-nlm-nih-gov.libproxy2.usc.edu/books/NBK44324/](https://www.zotero.org/google-docs/?0AOe1V)

[4. Vanker A, Gie R, Zar H. The association between environmental tobacco smoke exposure and childhood respiratory disease: a review. *Expert Review of Respiratory Medicine*. 2017;11(8):661-673. doi:10.1080/17476348.2017.1338949](https://www.zotero.org/google-docs/?0AOe1V)

[5. Wellman RJ, Dugas EN, Dutczak H, et al. Predictors of the Onset of Cigarette Smoking. *American Journal of Preventive Medicine*. 2016;51(5):767-778. doi:10.1016/j.amepre.2016.04.003](https://www.zotero.org/google-docs/?0AOe1V)

[6. Wiencke JK, Kelsey KT. Teen smoking, field cancerization, and a “critical period” hypothesis for lung cancer susceptibility. *Environmental Health Perspectives*. 2002;110(6):555-558. doi:10.1289/ehp.02110555](https://www.zotero.org/google-docs/?0AOe1V)

[7. Centers for Disease Control and Prevention. *The Health Consequences of Smoking—50 Years of Progress*. Centers for Disease Control and Prevention; 2014. https://www.cdc.gov/tobacco/data_statistics/sgr/50th-anniversary/index.htm](https://www.zotero.org/google-docs/?0AOe1V)

[8. Haghani A, Arpawong TE, Kim JK, Lewinger JP, Finch CE, Crimmins E. Female vulnerability to the effects of smoking on health outcomes in older people. *PLOS ONE*. 2020;15(6):e0234015. doi:10.1371/journal.pone.0234015](https://www.zotero.org/google-docs/?0AOe1V)

**Figure S1**

*Results for AdrenomedullinAdj*


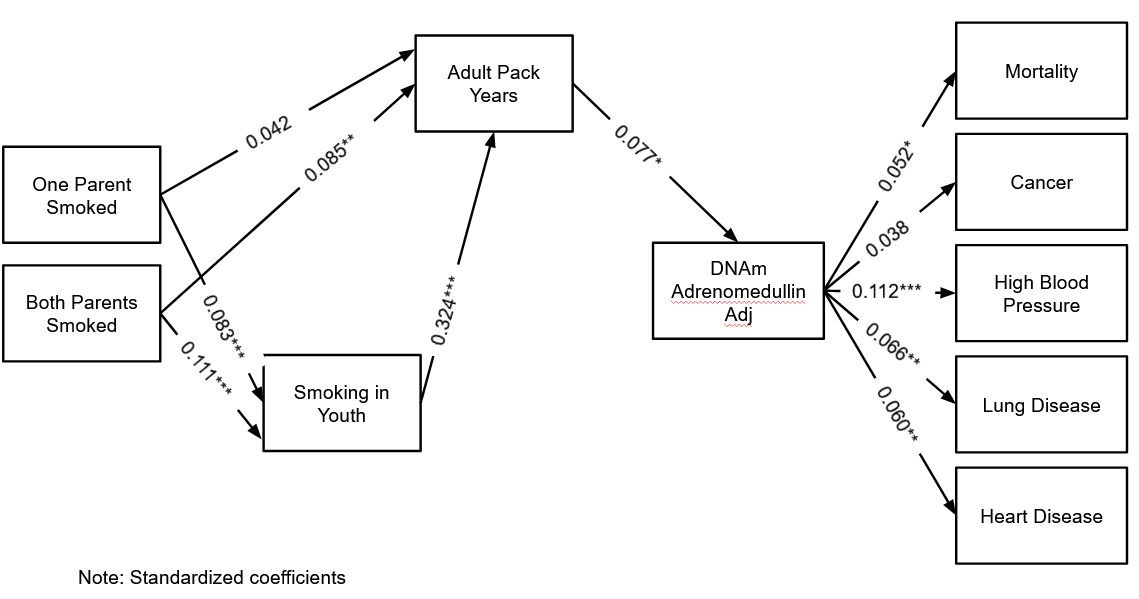


**Figure S2**

*Results for Beta-2-microglobulinAdj*


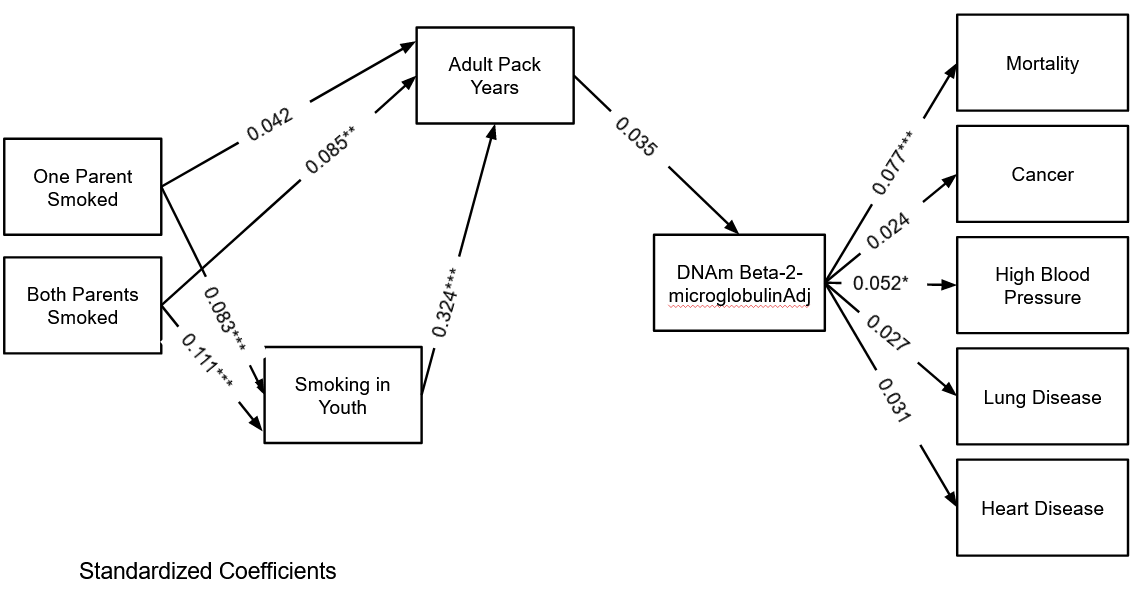


**Figure S3**

*Results for Cystatin-CAdj*


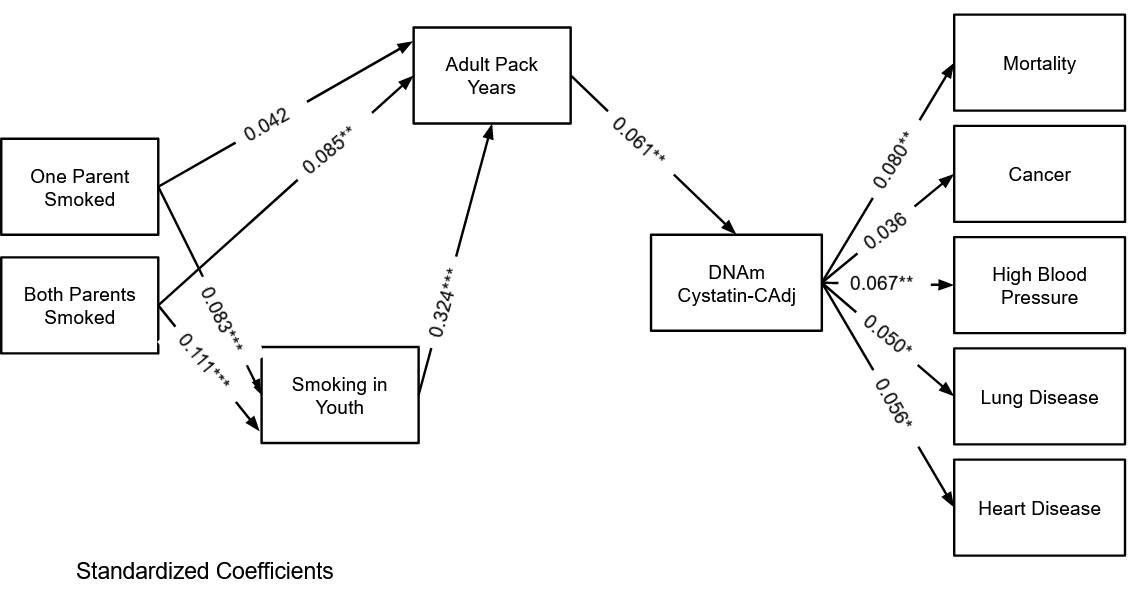


**Figure S4**

*Results for GDF-15Adj*


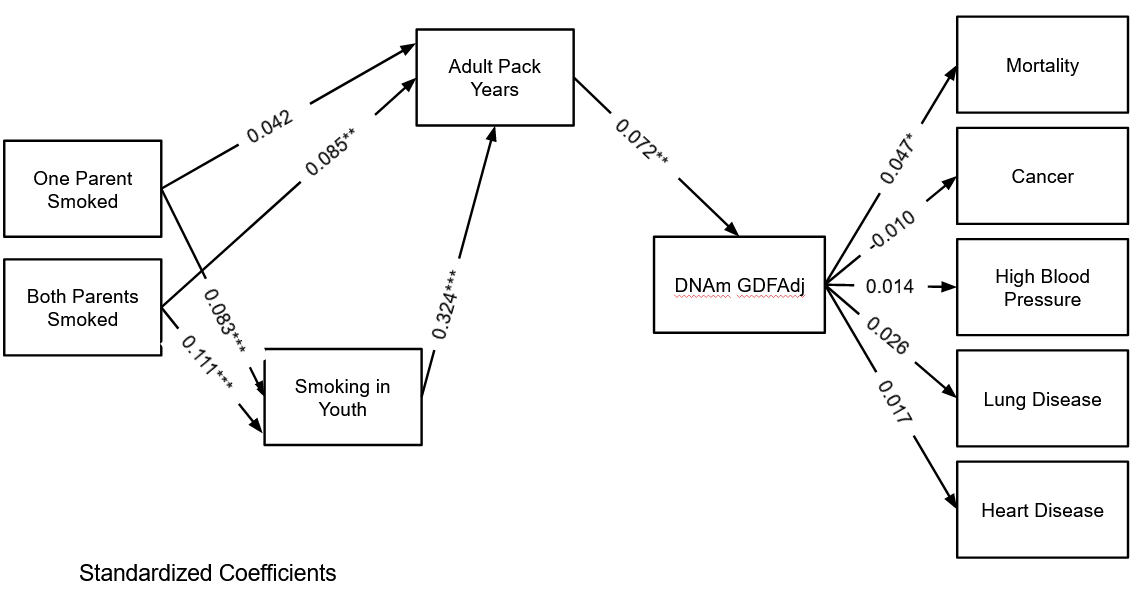


**Figure S5**

*Results for LeptinAdj*


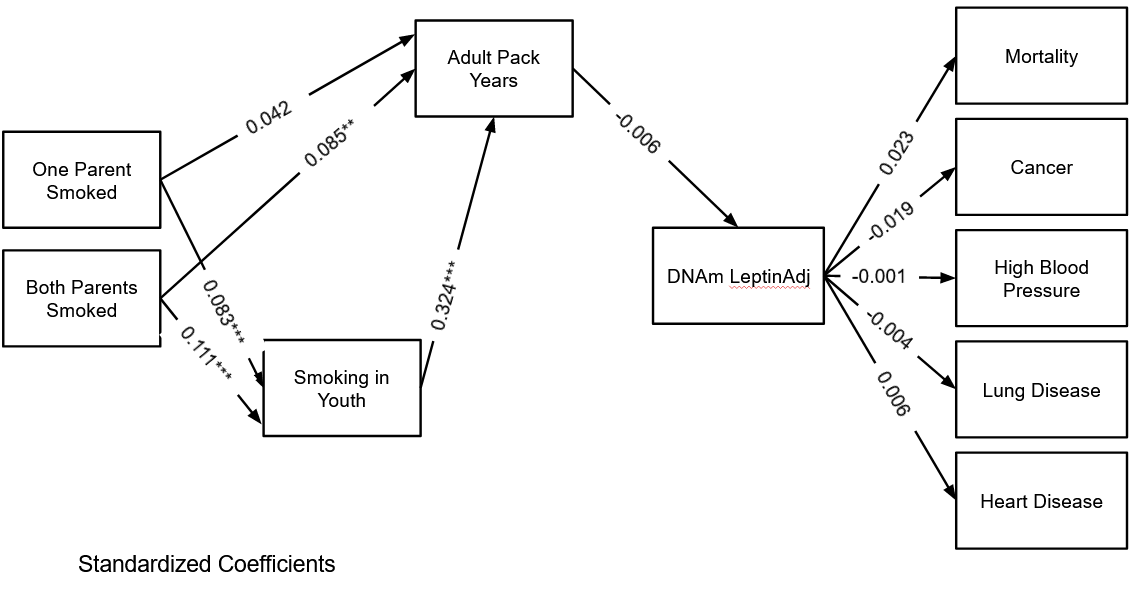


**Figure S6**

*Results for PackYearsAdj*


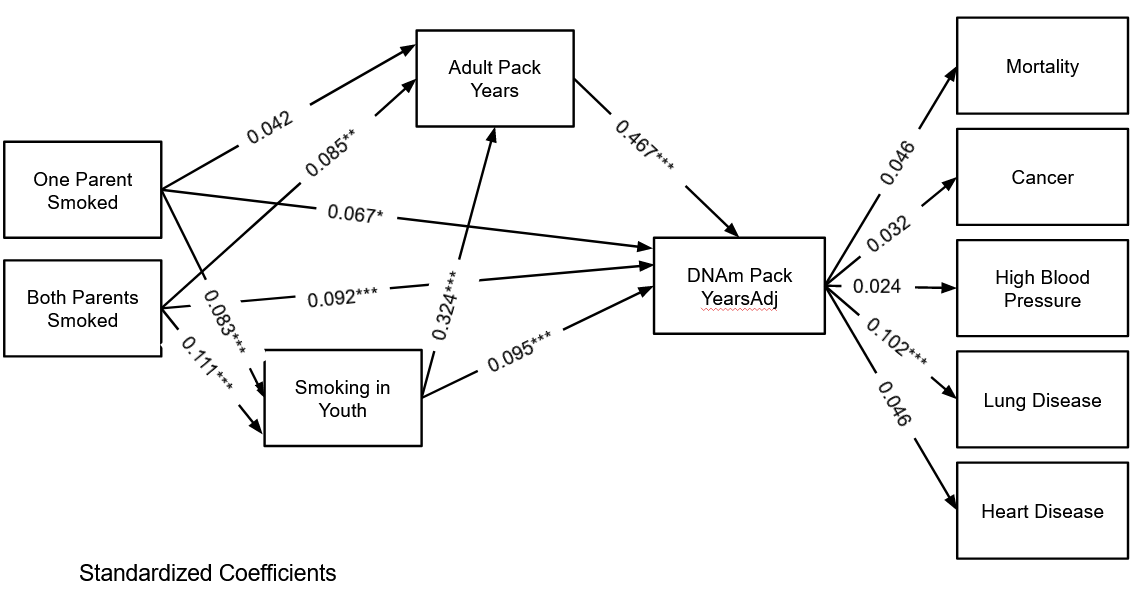


**Figure S7**

*Results for PAI-1Adj*


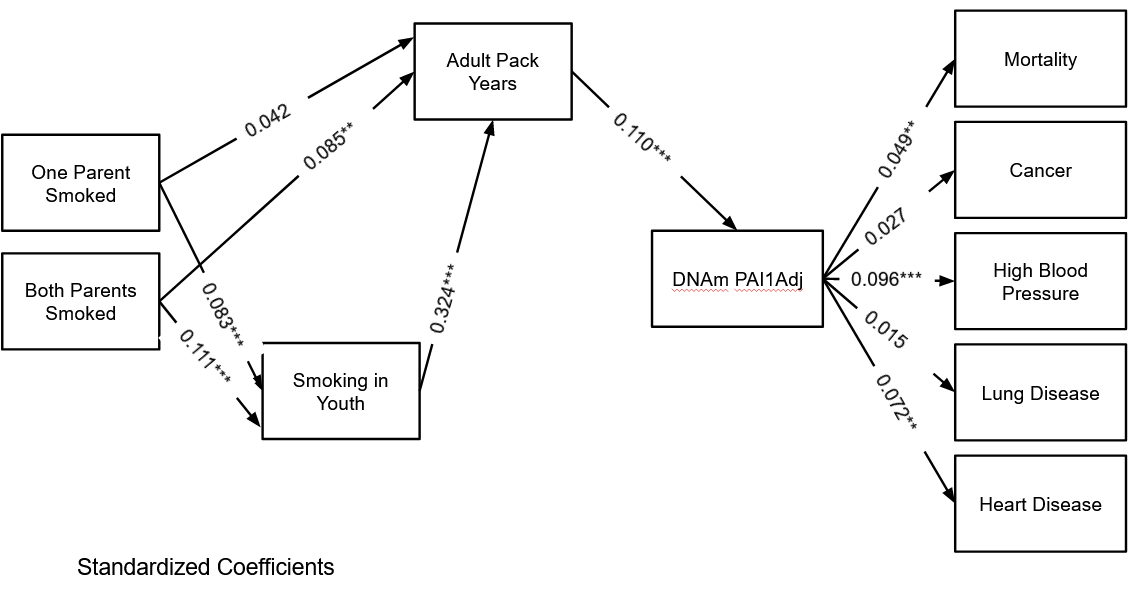


**Figure S8**

*Results for TIMP-1Adj*


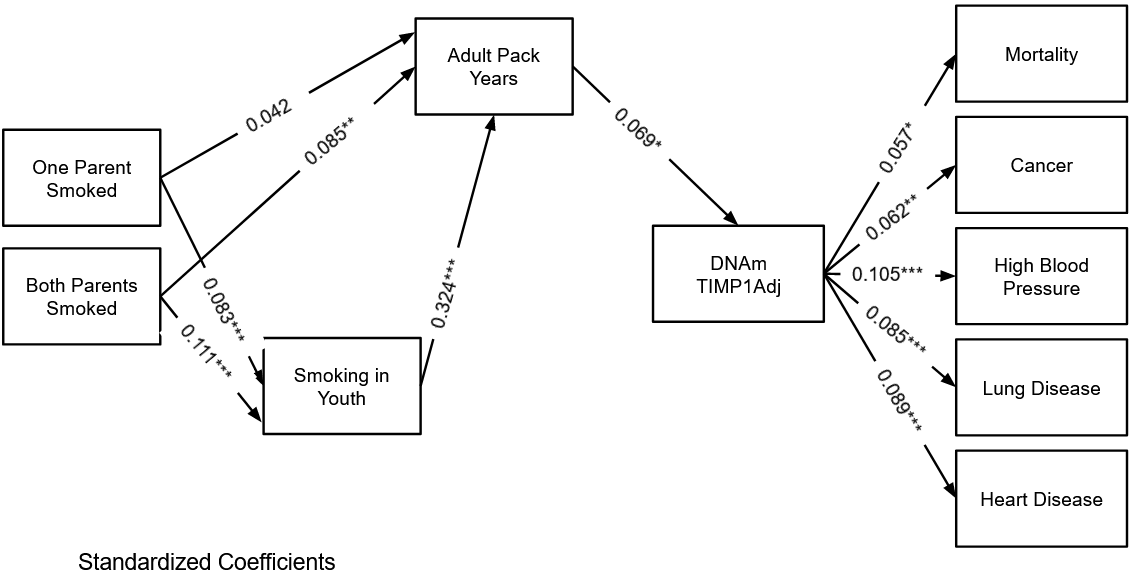

Supplement: Supplementary file 1 — Additional file 1: Supplementary Information. [file 13148_2022_1286_MOESM1_ESM.docx]
